# Pulmonary Embolism Presenting with Pulmonary Infarction: Update and Practical Review of Literature Data

**DOI:** 10.3390/jcm11164916

**Published:** 2022-08-21

**Authors:** Giulia Gagno, Laura Padoan, Stefano D’Errico, Elisa Baratella, Davide Radaelli, Alessandra Lucia Fluca, Alessandro Pierri, Milijana Janjusevic, Elena Aleksova Noveska, Maria Assunta Cova, Roberto Copetti, Franco Cominotto, Gianfranco Sinagra, Aneta Aleksova

**Affiliations:** 1 Cardiothoracovascular Department, Azienda Sanitaria Universitaria Giuliano Isontina (ASUGI) and Department of Medical Surgical and Health Science, University of Trieste, 34149 Trieste, Italy; 2 Cardiology and Cardiovascular Physiopathology, Azienda Ospedaliero-Universitaria S. Maria della Misericordia, 06156 Perugia, Italy; 3 Department of Medicine, Surgery and Health, University of Trieste, 34149 Trieste, Italy; 4 Department of Radiology, University of Trieste, ASUGI, Cattinara Hospital, 34149 Trieste, Italy; 5 Department of Pediatric and Preventive dentistry, Faculty of Dental Medicine, Ss. Cyril and Methodius University of Skopje, 1000 Skopje, North Macedonia; 6 Emergency Department, University Hospital and Health Services of Trieste, 34149 Trieste, Italy

**Keywords:** pulmonary infarction, pulmonary embolism, pulmonary ultrasound, diagnostic algorithm

## Abstract

Pulmonary infarction (PI) is a possible consequence of pulmonary embolism (PE). The real incidence of PI could be underestimated considering only non-fatal PE presentation. However, following postmortem examination, the prevalence of PI is considerably higher. This evidence suggests the necessity of proper diagnostic protocol for identifying PI. Unfortunately, PI diagnosis can sometimes be challenging, due to the overlapping of symptoms with other diseases. Nowadays, the diagnosis is mainly based on radiological evaluation, although the combination with emerging imaging techniques such as ultrasound and nuclear scanning might improve the diagnostic algorithm for PI. This review aims to summarize the available data on the prevalence of PI, the main predisposing factors for the development of PI among patients with PE, to resume the possible diagnostic tools, and finally the clinical and prognostic implications.

## 1. Introduction

Pulmonary infarction (PI) is the consequence of the blockage of distal pulmonary arteries, which results in an ischemic insult that further leads to necrosis of the obstructed pulmonary tissue [1]. PI commonly occurs due to complications of another existing disease, such as pulmonary embolism (PE) in most cases, but it can also occur in the context of various infections and malignancy [1]. Very often misdiagnosis or delayed diagnosis of PE results in difficult identification of PI [2,3]. Furthermore, the short- and long-term consequences after PE that can lead to PI and a higher risk of death are still unclear [1,4,5].

This paper aims to provide available data on the prevalence of PI among individuals with PE, which is the most common cause of PI, the possible diagnostic strategies, and the clinical and prognostic impact of this disease.

## 2. Epidemiology

Estimating the true prevalence of PI is difficult due to different definitions encountered in the literature [1]. Retrospective and prospective studies have assessed this knowledge gap [2,6,7,8]. For example, a retrospective study on 154 patients diagnosed with PE found that 29.2% had experienced PI, which was defined as a roughly homogenous region or a segment with “ground-glass opacity” on computed tomography (CT) examination [2]. Other studies also pointed out that PI is a possible complication of PE in more or less 30% of non-fatal cases [1,3]. However, PI should not be underestimated as its prevalence is much higher in postmortem examination of PE cases. A study by Mordeglia et al. that counted 560 autopsy examinations of patients with PE demonstrated that the prevalence of PI was 60%, which represent a very high number [9]. Therefore, when the death occurs unexpectedly or PE is suspected, an autopsy should be considered mandatory to investigate the PI [5].

Given the relevant incidence at forensic examinations, efforts have been made to identify predisposing factors for PI after PE. Trying to elucidate the predictors of PI in young patients with PE, Miniati et al. have reported that the odds ratio (OR) with a 95% confidence interval was 3.6 (1.88–6.91) for cigarette smoking, 1.16 (1.05–1.29) for age, and 1.04 (1.01–1.07) for body height [7]. Some observations indicate that individuals with other cardiopulmonary comorbidity have a higher risk of PI after acute PE [1]. Although consistent with the previous definition of PI, Kirchner et al. found no correlation between the risk of PI and heart failure, malignancy, clot burden, or preexisting pulmonary infections [2]. There is consensus that hemodynamic instability is a factor associated with adverse outcomes [10] but the assessment of the patient’s hemodynamic status in increasing PI incidence was beyond the scope of the Kirchner et al. study [2]. In particular, there are numerous findings indicating that the frequency of PI after PE is higher in patients who died from long-standing heart failure [7]. However, data on this field for young and healthy individuals are scarce and conflicting. From what has been described so far [2], efforts are needed for the stratification of patients who may develop PI.

## 3. Physiopathology

Although not all cases of PE lead to PI, the relevant percentage of PI diagnosis on autopsy suggests the importance of understanding its pathogenesis as a first step toward a correct diagnosis.

In about 90% of cases, pulmonary artery occlusion is caused by thrombus formation in veins of the lower limb [11]. In absence of deep vein thrombosis, pulmonary artery occlusion could be explained by in situ thrombi formation [12]. When a pulmonary artery is occluded, the pressure in bronchial arteries increases to perfuse pulmonary capillaries [1]. Impaired blood flow and endothelial cell ischemia contribute to the increase in capillary permeability, which facilitates the extravasation of erythrocytes and causes alveolar hemorrhage [1]. Patients with impaired hemodynamics are more prone to PI since it interferes with the resolution of alveolar hemorrhage thus worsening necrosis [13]. This explains the increased risk of adverse outcomes in patients with hemodynamic instability [11]. These processes are reversible in some cases defined as incomplete infractions; the blood from the intra-alveoli can be reabsorbed within 2–4 days without causing necrosis of the surrounding tissue. On the other hand, in cases defined as true infarction when blood is not absorbed, tissue necrosis occurs within 1–2 days due to the destruction of erythrocytes to hemosiderin and alveolar bleeding [1,14]. After the insult, the formation of a fibrous scar occurs in the area affected by the infarct for a period of several weeks or even months [1].

PI is usually subpleural and wedge-shaped grossly and, on microscopic examination, appears as a region of dead alveolar walls. From a histological point of view, PI is defined as a well-defined area of coagulative necrosis of the lung parenchyma within a zone of hemorrhage, usually adjacent to the pleura [6]. Coagulative necrosis is a type of necrosis defined as a morphological feature in which protein denaturation is the main process coupled with minor enzymatic degradation, which consequently leads to longer preservation of tissue architecture after cell death. In the insulted area, there are usually present the metaplastic and alveolar atypical epithelial cells; and the surrounding pleural surface is commonly covered with fibrinous transude. Finally, thrombi are not always detectable histologically [1].

Localization of emboli on distal pulmonary arteries is a common characteristic of PI [8], which could occur even in young and healthy individuals [7]. Autopsy studies indicate that the diameter of vessels occluded by emboli could not be the major factor in determining PI. Specifically, the extension of the occlusion from arteries larger than three millimeters in diameter to smaller distal branches was not associated with PI. As opposed to emboli in pulmonary arteries of three or fewer millimeters in diameter, which are localized with PI [14]. Several studies have reported a trend in the localization of PI in the lower lobe but is not yet clear whether this is due to hemodynamic factors or not [2]. Furthermore, PI appears to be more likely when PE is determined by the occlusion of subsegmental pulmonary arteries [15].

## 4. Possible Diagnostic Criteria

### 4.1. Signs and Symptoms of Pulmonary Infarction

The diagnosis of PI is often challenging due to the limited specificity of signs and symptoms [16]. Therefore, PI is very likely to be underdiagnosed from a clinical perspective. Besides this, recognizing PI plays an important role in optimizing the management of patients affected by PE [16].

Unfortunately, many symptoms such as dyspnea, weakness, dizziness, and syncope, as well as chest wall tenderness, tachypnea, and tachycardia, are not strictly related to PI making differential diagnosis even more difficult [3]. Nonetheless, patients with PI are more likely to present with pleuritic chest pain [2] and its association with hemoptysis and fever furtherly increases the clinical suspicion of this entity [16]. PI is compatible with pneumonia, chronic obstructive pulmonary disease, and congestive heart failure [4]. Since radiographic imaging may be unreliable and ambiguous, there is a high risk of the wrong conclusion that the cause of the patient’s symptoms is one of these entities instead of PI [4,17]. Furthermore, when approaching an otherwise young and healthy individual with unexplained pleuritic chest pain or hemoptysis in the emergency department, a PI complicating an acute PE should be considered a possible diagnosis among other conditions (e.g., pneumonia, lung neoplasia, lung granulomatous disease) [3,4].

### 4.2. Imaging Techniques for the Diagnosis of Pulmonary Infarction

Since PE is a potentially fatal condition that requires prompt intervention, it has to be ruled out with the most appropriate diagnostic methods as described by current guidelines [10]. This paragraph discusses whether approved or suggested methods for diagnosing PE can be promising and valid in the identification of PI.

**Figure 1:**
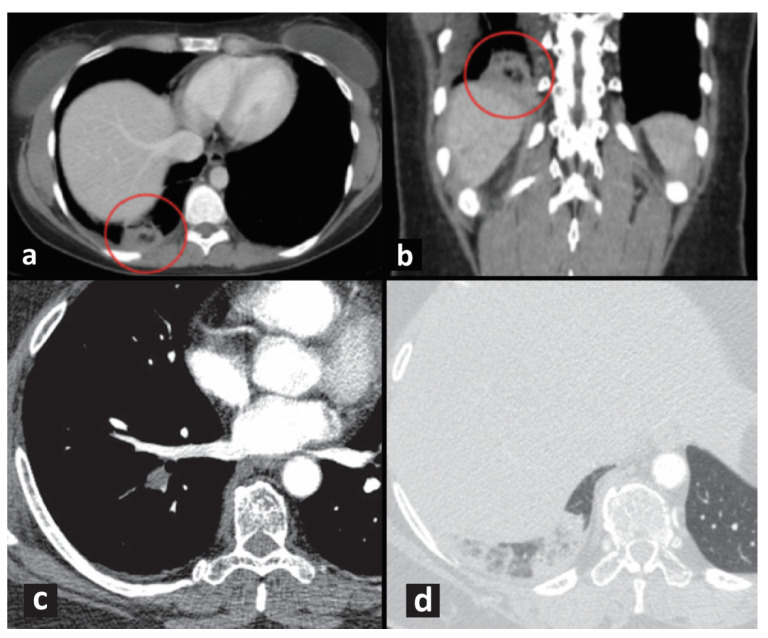
(**a**,**b**) CT of typical “bubbly consolidation” with central lucencies, indicated by red circles. (**c**) CT pulmonary angiography shows a filling defect within a segmental artery of the right lower lobe (acute pulmonary embolism). (**d**) Typical findings of a pulmonary infarction are better recognizable on images reconstructed with a parenchymal filter: a peripheral pleural-based consolidation with convex borders (Hampton hump) and small central clearance, without air bronchogram in the lower right lobe.

Nowadays, the diagnosis of PE is mainly based on radiomics, which has also been evaluated for the diagnosis of PI [18,19]. In particular, CT pulmonary angiography (CTPA) is the gold standard with high accuracy [20] and dual-energy CTPA allows the identification of perfusion defects [20,21]. Acute PE could cause partial or complete obstruction of pulmonary vasculature but the latter can lead to PI, which appears on CTPA as ground-glass opacity with reticular changes or wedge-shaped consolidation with its base toward the pleural surface (Hampton hump) and truncated apex toward the hilum, without air bronchogram [7,20,22,23]. A central lucency in peripheral consolidation, defined as “bubbly consolidation” suggests PI. Moreover, diffused or localized pleural effusion and thickened vessels are other salient findings of PI at CTPA examination [23]. Moreover, complete occlusions appear tortuous and calcified [20]. It is important to consider the presence of artifacts due to breathing, which could lead to misdiagnosis [22,24] (Figure 1).

In some cases, multi-slice CTPA (MCTPA) has been provided as a valid approach to detect emboli [21,25] and PI [26]. In MCTPA, PI has been described as triangular (wedge-shaped) opacity with sharp margins consisting of focal radiolucency or “bubbly consolidations”, which is considered a specific marker for PI [27,28].

**Figure 2:**
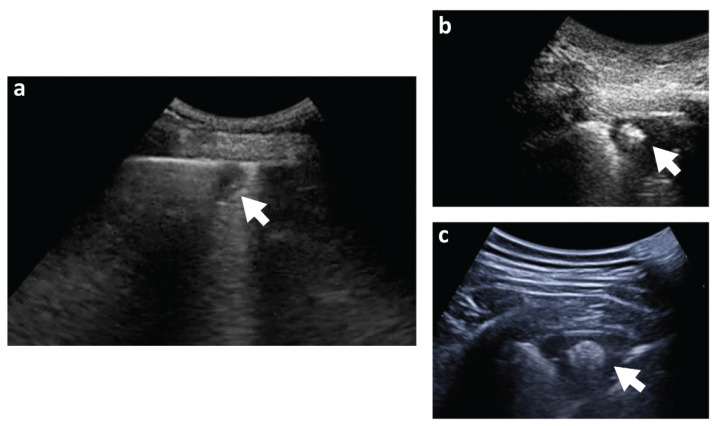
(**a**) Small (11 × 9 mm) subpleural consolidation detected at lung echography. Clear pleural discontinuity with the underlying hypoechoic area. (**b**,**c**) Typical ultrasound appearance of “bubbly consolidation” with a roundish hyperechoic area in the context. Arrows indicate the consolidation at lung echography.

The “bubbly consolidations” seem to correspond to a central rounded hyperechoic area in the absence of bronchograms at lung echography [29] (Figure 2). Ultrasound (US) is a routine diagnostic tool in patients with dyspnea and/or chest pain in daily clinical practice, especially in the emergency setting, and the method is also reproducible and radiation-free [30]. Although not mentioned in the current ESC guidelines, recent studies have confirmed the validity of pulmonary US in patients with PE [31,32,33,34]. Also, PI can be visualized with the US [35]. Mathis et al. demonstrated that in a large cohort of patients, pulmonary US was able to detect pleural effusion and subpleural pulmonary consolidation consistent with PI in patients with PE [23,36]. Pulmonary US has a potential role as a supporting diagnostic tool in individuals with suspected PI who should undergo CTPA but for various reasons are not candidates [37]. Interestingly, in one study, contrast-enhanced ultrasound was informative for PI diagnosis in patients with PE and negative CTPA [38]. Despite this promising data, pulmonary US is characterized by some limitations when used alone [32] as it can miss central lesions and is able to identify the alterations related to the so-called “early infarction” or “pulmonary hemorrhage” on a narrow time frame (a few hours) [37]. Moreover, this technique is influenced by the examiner, is time-consuming, and requires practice [39].

Currently, CTPA remains the gold standard for the diagnosis of PE as well as for PI and its complications [23]. However, results of a meta-analysis have suggested that the specificity and sensitivity of Magnetic Resonance Imaging (MRI) are slightly better than CTPA ones for the detection of acute PE [40]. Furthermore, MRI successfully identified PI on post-mortem examination [41]. Therefore, MRI could be useful when CTPA is insufficient for the diagnosis of PI, thus helping in the differential diagnosis of alternative causes of parenchymal opacity [40]. Furthermore, MRI could discriminate the time of occurrence of PI. In particular, the lesions have low signal intensity on T1-weighted and high signal intensity on T2-weighted images within the first 24 h [41]. While in the subacute phase, the lesions are characterized by a hyperintense signal on both T1- and T2-weighted imaging [41]. Through MRI it is possible to perform a functional and anatomical evaluation of the vascular system and lung tissue avoiding exposure to radiation or iodinated contrast media [23]. For these reasons, it can be a useful technique in case of contraindications to CT, with good sensibility and specificity [42]. However, this technique is usually not readily available in the acute care setting, so its use is often limited in clinical practice.

### 4.3. Molecular Diagnosis

In current guidelines, D-dimer is the only biomarker for diagnosis of PE while Troponin or brain natriuretic peptides (BNPs) are the election biomarkers for assessing the outcome [10]. However, the usefulness of these biomarkers for diagnosis and prognosis appears limited when used alone as they are valuable to identify other pathologies such as myocardial infarction and heart failure. Therefore, they are used in combination with imaging techniques [43]. The increase in Troponin or BNPs concentration could arise as a consequence of embolism [10,43,44]. However, the literature lacks studies evaluating the use of biomarkers in the context of PI diagnosis [43]. Similarly, the increase in inflammatory biomarkers, which could be secondary to numerous other pathological conditions, is not particularly informative but opens prospects for prognostic evaluation in the context of PI [45,46].

## 5. Clinical Management and Prognostic Implication

The impact of PI following acute PE and the prognosis after specific management strategies are still not clear [1]. Thus, further studies to clarify these aspects are needed, to develop a patient-tailored protocol and to reassure the patients’ recovery. Indeed, studies on PI among PE patients concentrate on endpoints defined as the PI incidence, PE recurrence, cavitation, infectious complications, and death. Further research is needed to focus on the missing data such as the incidence of the post-PE syndrome and more importantly, novel therapeutic approaches [1]. Currently, there are no specific therapeutic strategies for PI, except for supportive measures and pain control with non-steroidal anti-inflammatory drugs or opioids [1,47]. Furthermore, PI can be complicated by lung infections that are difficult to control, leading to abscess formation and cavitation, which are related to various predisposing factors such as more extensive infarction, concomitant congestion, or atelectasis, and poor dental health [1]. For this reason, the initiation of empiric antibiotic therapy has been proposed as a reasonable therapeutic strategy in case of PI detection, to avoid further complications such as empyema or fistulae, which may require surgical therapy [48].

Due to the impact of PI on prognosis, its presence should be taken into account when stratifying the risk of patients with PE. This necessity is even more urgent in young patients and otherwise healthy individuals as misdiagnosis of PI in this group appears critical [4,10]. Since the infarcted area is ideal for the development of infections and inflammation, it could be responsible for adverse outcomes in young individuals [1,49].

## 6. Pulmonary Infarction: What Are the Prospects for the Diagnostic Algorithm?

Based on the evaluation of some clinical cases available in the literature, PI still represents a diagnostic challenge with a negative impact on the survival rate in case of misdetection [48,50]. In this section, we summarize how diagnostic progress can translate into a management strategy for patients with PI.

**Figure 3:**
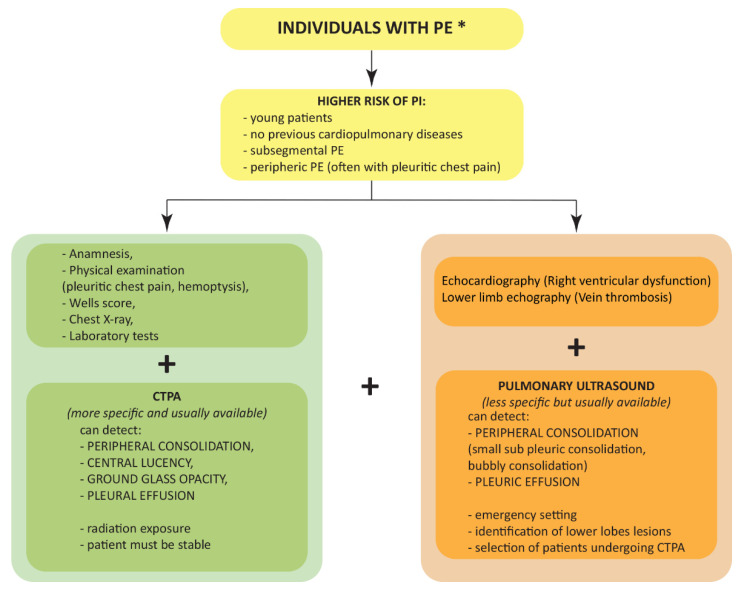
Flow diagram showing the proposed algorithm for the diagnosis of pulmonary infarction in case of pulmonary embolism. * As diagnosed via current guidelines. CTPA, computed tomography pulmonary angiography; PE, pulmonary embolism; PI, pulmonary infarction.

The Wells and Geneva scores are used to rank individuals based on the probability of acute PE and diagnostic tests are chosen based on these scores [51]. Therefore, patients with a high probability of thromboembolism are likely to be easily diagnosed with PI through the use of the same diagnostic techniques [50,51]. Although the CTPA remains the gold standard for the diagnosis of PI, in some cases this diagnostic method is not sufficient [10]. Therefore, other techniques have been proposed in this review. Improving diagnostic algorithm supporting CTPA with pulmonary US evaluation or MRI, especially in the emergency setting when patients cannot be easily transported, is not immediately feasible. However, there are prospects for improvement, as demonstrated by the current growing diffusion of pulmonary US in different clinical settings. A recent meta-analysis estimated that the US has a sensitivity of 91% and a specificity of 81% for the diagnosis of PE when compared with CTPA [33]. Pulmonary US and MRI exams should not replace the validated diagnostic tools for PE but represent additional imaging modalities when PI is suspected (Figure 3) [39]. The main problem in the diagnosis of PI is the high variability in the time of presentation after PE. Therefore, the absence of PI at the diagnosis of PE does not rule out later development. In this case, follow-up checks could be the keys to the timely diagnosis of PI.

Once PI is diagnosed, the therapeutic strategy should be aimed at treating the underlying cause and lowering the risk of its serious complications. In this regard, oxygen administration can be useful to prevent hypoxia [10] as well as antibiotic therapy should be considered to avoid infectious complications [48]. Whatever PE is the cause of PI, pharmacological treatment includes anticoagulant medication such as heparin followed by warfarin or other oral anticoagulants [10,16]. In case of coexistence of hemodynamic instability, the administration of systemic fibrinolytic or reperfusion treatments is approved [10,16].

## 7. Conclusions

Increasing our knowledge of the prevalence of PI among individuals with PE, especially young ones, together with the awareness of the most commonly associated risk factors could lead to a correct stratification of patients with PI, which emerges as a tremendous achievement. Moreover, new and highly sensitive diagnostic tools will have a major impact on our understanding of this pathology with further important implications for clinical practice and treatment.

## Data Availability

Not applicable.
